# Exemplary post-discharge stroke rehabilitation programs: A multiple case study

**DOI:** 10.1177/02692155221144891

**Published:** 2022-12-21

**Authors:** Mary Egan, Debbie Laliberte Rudman, Monique Lanoix, Matthew Meyer, Elizabeth Linkewich, Phyllis Montgomery, Jenn Fearn, Beth Donnelly, Margo Collver, Shauna Daly

**Affiliations:** 1 School of Rehabilitation Sciences, University of Ottawa, Ottawa, Ontario, Canada; 2 School of Occupational Therapy, 70383 Western University Faculty of Health Sciences, London, Ontario, Canada; 3 Faculty of Philosophy, 10064 Saint Paul University, Ottawa, Ontario, Canada; 4 Senior Leadership, 10033 London Health Sciences Centre, London, Ontario, Canada; 5 Regional Stroke Centre, 71545 Sunnybrook Health Sciences Centre, Toronto, Ontario, Canada; 6 School of Nursing and Allied Health Professions, 7728 Laurentian University, Sudbury, Ontario, Canada; 7 Northeastern Stroke Network, Health Sciences North, Sudbury, Ontario, Canada; 8 Champlain Regional Stroke Network, The Ottawa Hospital, Ottawa, Ontario, Canada; 9 Regional Stroke Centre, 10033 London Health Sciences Centre, London, Ontario, Canada; 10 Rehabilitation, 25474 Bruyere Continuing Care, Ottawa, Ontario, Canada

**Keywords:** Patient-centered care, qualitative study, stroke, team

## Abstract

**Objective:**

The objective of this study was to identify essential aspects of exemplary post-discharge stroke rehabilitation as perceived by patients, care partners, rehabilitation providers, and administrators.

**Design:**

We carried out an exploratory qualitative, multiple case study. Stroke network representatives from four regions of the province of Ontario, Canada each nominated one post-discharge rehabilitation program they felt was exemplary.

**Setting:**

The programs included: a mixed home- and clinic-based service; a home-based service; a clinic-based service with a stroke community navigator and; an out-patient clinic-based service.

**Participants:**

Participants included 32 patients, 16 of their care partners, 23 providers, and 5 administrators.

**Methods:**

We carried out semi-structured qualitative interviews with patients and care partners, focus groups with providers, and semi-structured interviews with administrators. Health records of patient participants were reviewed. Using an interpretivist-informed inductive content analysis, we developed overarching categories and subcategories first for each program and then across programs.

**Results:**

Across four regions with differing types of programs, exemplary care was characterized by three essential components: stroke and stroke rehabilitation knowledge, relationship built through personalized respectful care, and a commitment to high quality, person-centered care.

**Conclusion:**

Exemplary post-discharge care included knowledge regarding identification and treatment of stroke-related impairment, that is, information found in best practice guidelines. However, expertise related to building relationship through providing personalized respectful care, within a mutually supportive, improvement-oriented team was also essential. Additionally, administrators played a crucial role in ensuring continued ability to deliver exemplary care.

## Introduction

Approximately half of people hospitalized due to stroke could benefit from rehabilitation following discharge to address continuing issues with daily life and social roles.^1^ While the processes of in-patient stroke rehabilitation have been well documented,^2,3^ post-discharge or community-based stroke rehabilitation has been the subject of less study. Post-discharge stroke rehabilitation differs from in-patient care in that it occurs in a variety of settings, including outpatient clinics and patient homes. As well, when funding limits are not an issue, there is often no clear marker for when service should end.^4^ Given that both features raise additional possibilities and considerations for care, greater understanding of post-discharge stroke rehabilitation is warranted.

The Canadian best practice guidelines state that post-discharge stroke rehabilitation should be as similar as possible to in-patient rehabilitation.^5^ However, for several reasons, it may not be feasible for post-discharge stroke rehabilitation to mirror in-patient care, particularly in terms of intensity. First, patient factors may limit such application. For example, a patient may be too fatigued to manage intensive programming.^6^ Second, modifications may be required for demographic reasons. For example, a region may not have adequate numbers of therapists to deliver intensive therapy.^7^ Third, when the focus is on intensity, therapy resources may be exhausted before meaningful, sustainable participation outcomes are attained. Patients may feel abandoned when care ceases before they are able to re-engage in valued activities and social roles.^8^ Examination of a variety of post-discharge stroke rehabilitation programs perceived as exemplary could help identify features essential to such care that cut across patients and settings, while clarifying features that may differ from in-patient rehabilitation.

In Canada, stroke care is part of the publicly funded healthcare system that ensures access to physician services and in-patient hospital care. Provision of healthcare is a provincial responsibility, and public access to post-discharge rehabilitation varies from province to province. In the province of Ontario, process and funding directives framed as Quality Based Procedures guide stroke care.^9^ These recommendations represent the provincial interpretation of stroke best practice guidelines and other evidence, with directives outlining the amount of therapy to be provided. This helps ensure that, in the face of funding constraints, most patients receive a reasonable amount of service.

Within the Quality Based Procedures, standards for post-discharge rehabilitation are described under sections entitled General Rehabilitation and Core Team Recommended Practices. These sections characterize ideal post-discharge rehabilitation as services that remediate deficits, begin as soon as possible after hospital discharge, and are provided by a specialized team, ideally in a centralized location.^9^ Regardless of location, providers are directed to offer, on average, 2–3 visits per week over 8–12 weeks.^9^

Recommendations for local application of Quality Based Procedures are made by 11 regional stroke networks, who take into consideration unique regional needs. Post-discharge rehabilitation services are designed and provided through regional home care programs and hospitals. This results in varied programs that aim to deliver best practice within local contexts. The objective of this study was to identify essential aspects of exemplary post-discharge stroke rehabilitation as perceived by patients, care partners, rehabilitation providers, and administrators through a close examination of exemplary post-discharge rehabilitation programs across four distinct regions of Ontario, Canada.

## Method

We carried out a multiple case study,^10^ using an exploratory qualitative approach^11^ consistent with a subjectivist epistemology and a relativist ontology.^12^ This study formed part of a grant exploring the design of post-discharge rehabilitation as viewed from an ethics of care lens.^13^ This provided a particular focus on how patient and care provider needs were attended to and addressed, within the structure and resources available. The team was led by an occupational therapist/epidemiologist. Other team members had backgrounds in physiotherapy, occupational therapy, nursing, counselling, administration, epidemiology, and philosophy; most had extensive experience in stroke rehabilitation, stroke rehabilitation health services administration or stroke rehabilitation research.

We planned to study four cases—that is, four exemplary post-discharge stroke rehabilitation programs in Ontario, Canada. Ontario covers approximately 1 million square kilometers. With approximately 14 million people, it is Canada's most populous province. We did not aim to capture all possibilities but rather to ensure diversity in contextual and regional considerations. We selected our cases to provide a range of urban-rural composition, age distribution, and proportion of English and French-speakers. Representatives from the stroke networks of each of the four regions nominated one program that they considered exemplary. An exemplary case was defined as a program that was patient-centered and effective in helping patients return to valued activities. These joint aims reflect the statements in provincial guideline documents^9^ and the importance stroke survivors place on participation outcomes.^14^ Implicit in any nomination would be the provision of evidence-based care, as such care is championed and seen as necessary to exemplary care by all provincial stroke networks.

For each case, we gathered and analyzed multiple sources of data: program documents, patient and care partner interviews, provider focus groups, and administrator interviews. First, program documents, such as program descriptions on public websites, were reviewed to gain an initial understanding of the structure and process of the service. Then, eight people who had recently completed at least two months of service were identified by program staff and invited to participate by the research team. These service users were sampled purposefully^15^ to ensure inclusion of both men and women, older and younger patients, and people who lived with partners or alone. Patient participants were invited to identify a significant support person. Patient participants, and their care partners where present, participated in semi-structured qualitative interviews carried out by research assistants. Patients and care partners were asked to discuss their experience with the program, and how the program had and had not met their needs.

All interviews were audiorecorded and transcribed verbatim. Analysis was carried out by the first author and the research coordinator. Qualitative research software (NVivo 12) was used to help with the organization of the analysis. Consistent with interpretive description, the analysis strategy was developed to ensure results addressed the study objective.^16^ We began with the patient and care partner interviews, inductively identifying codes for aspects of care identified as important to them. These codes were then organized into categories and subcategories.

Next, focus groups were carried out with the programs’ rehabilitation providers. All providers were invited by the researchers to attend the focus groups. In this invitation we noted that we were hoping to have at least one provider per profession included in the program. Open-ended, discussion-based focus groups were led by the first author with help from the research coordinator or a research assistant. During each focus group, participants were asked first to discuss how they felt their program was exemplary. Then, the categories of exemplariness derived from patient participant interviews were shared with the providers. Providers were asked how their program functioned to produce these characteristics and whether they saw any potential issues that could affect their ability to continue to carry out the program in ways that were exemplary.

Subsequently, individual semi-structured qualitative interviews were carried out with program administrators. Similarly to the healthcare provider interviews, we began by asking administrator participants how they felt the program was exemplary. We then shared the characteristics that patient participants identified and asked the administrators how the program demonstrated these characteristics, how these characteristics were maintained, and whether the administrators felt there were any threats to the program's ability to continue to operate in this way. Audiorecordings of all interviews and focus groups were transcribed verbatim.

Finally, patient participant program health records were reviewed in the first three regions (Programs A, B, and C); COVID-19 restrictions in place at the time did not allow us to carry out an in-person visit required for review of records in the fourth region (Program D). Instead, we met virtually with an administrator who described the format of the records and answered our questions regarding contents. During record review we identified how providers documented care and, particularly, where they referred to any categories of exemplary care as identified by the service users. All data was collected between September 2018 and August 2020.

Consistent with guidance for multiple case studies,^10^ the data for each region were analyzed separately. As noted above, data analysis began with coding of the patient interviews prior to focus groups with providers and interviews with administrators. Data from the provider and administrator interviews were coded inductively by the first author and the research coordinator, using codes from the analysis of patient and care partner participant interviews or new codes where necessary. After this, overarching categories and subcategories were determined through iterative sorting of the codes. Then, the categories were reviewed a final time to ensure a complete set of relatively discrete categories related to exemplary care. For each region, categories, subcategories, and illustrations from the data were shared with a program administrator in the form of an overall report. Administrator feedback was used to further refine each single case analysis.

Consistent with recommendations for analyzing data in multiple case studies,^10^ in the cross-case analysis, we began with the results of the analysis of one program, in this case, Program A. We then compared categories and subcategories from Program B, making refinements and additions as necessary to provide an overarching picture of exemplary rehabilitation. We continued in this manner until the results took into consideration the information from all four programs. Finally, the connections across the shared cross-case categories and the actions of providers and administrators were aligned to develop a framework describing actions involved in exemplary care and how these actions worked together to support stroke recovery.

Trustworthiness checks were included at each stage of data collection. That is, at each data collection point we reviewed with participants what we had learned previously. As noted above, overall results of the analysis for each region were refined with feedback from an administrator from the region.

Reviews were completed by the ethics committees of Bruyere Continuing Care (16-17-050), the University of Ottawa (H-01-18-286), Western University (111696), and University Health Network (18-5395).

## Results

### Programs and Contexts

Characteristics of the regions from which programs were selected are outlined in Table 1. The regions demonstrated variability in urban/rural mix, age distribution, and proportion of rehabilitation providers by population (Supplementary Material). The programs included a clinic-based program (Program D), a home-based program (Program A), and two programs with a mix of both clinic and home visits (Programs B and C). Service provision varied, but typically included occupational therapy, physiotherapy, speech-language pathology, social work, and rehabilitation assistance. Program C had a unique provider model that included a Stroke Community Navigator. In this program, an occupational therapist and a physiotherapist were contracted part-time; they assessed patients and provided advice regarding individual programming that was carried out by rehabilitation assistants.

**Table 1.** Region characteristics.

Program	A	B	C	D
Region type	Small city with much of population in surrounding rural areas	Medium-sized city with some of population in surrounding rural areas	Small northern (more remote) city with much of population in surrounding small towns	Large city
Proportion population rural (%)	60	28	30	0

Additional characteristics in supplementary material.

Participant characteristics are outlined in Table 2. Patient participants ranged in age from forties to eighties. Most care partner participants were spouses. Provider participants included participants from each of the professions included in the program.

**Table 2.** Participants.

Program	A	B	C	D
Patients	4 men, 4 women Aged 45–86	4 men, 4 women Aged 54–78	6 men, 2 women Aged 41–78	6 men, 2 women
Care partners	3 spouses	3 spouses	5 spouses 1 daughter 1 mother 1 friend	2 spouses
Providers	7 providers including individuals from Occupational therapy Physiotherapy Speech-language pathology Social work Rehabilitation assistant	6 providers including individuals from Occupational therapy Physiotherapy Speech-language pathology Social work Rehabilitation assistant	2 providers: Stroke community navigator Rehabilitation assistant	8 providers including individuals from Occupational therapy Physiotherapy Speech-language pathology Social work
Administrators	2 administrators	1 administrator	1 administrator	1 administrator

### Exemplary Care

**Figure 1.**
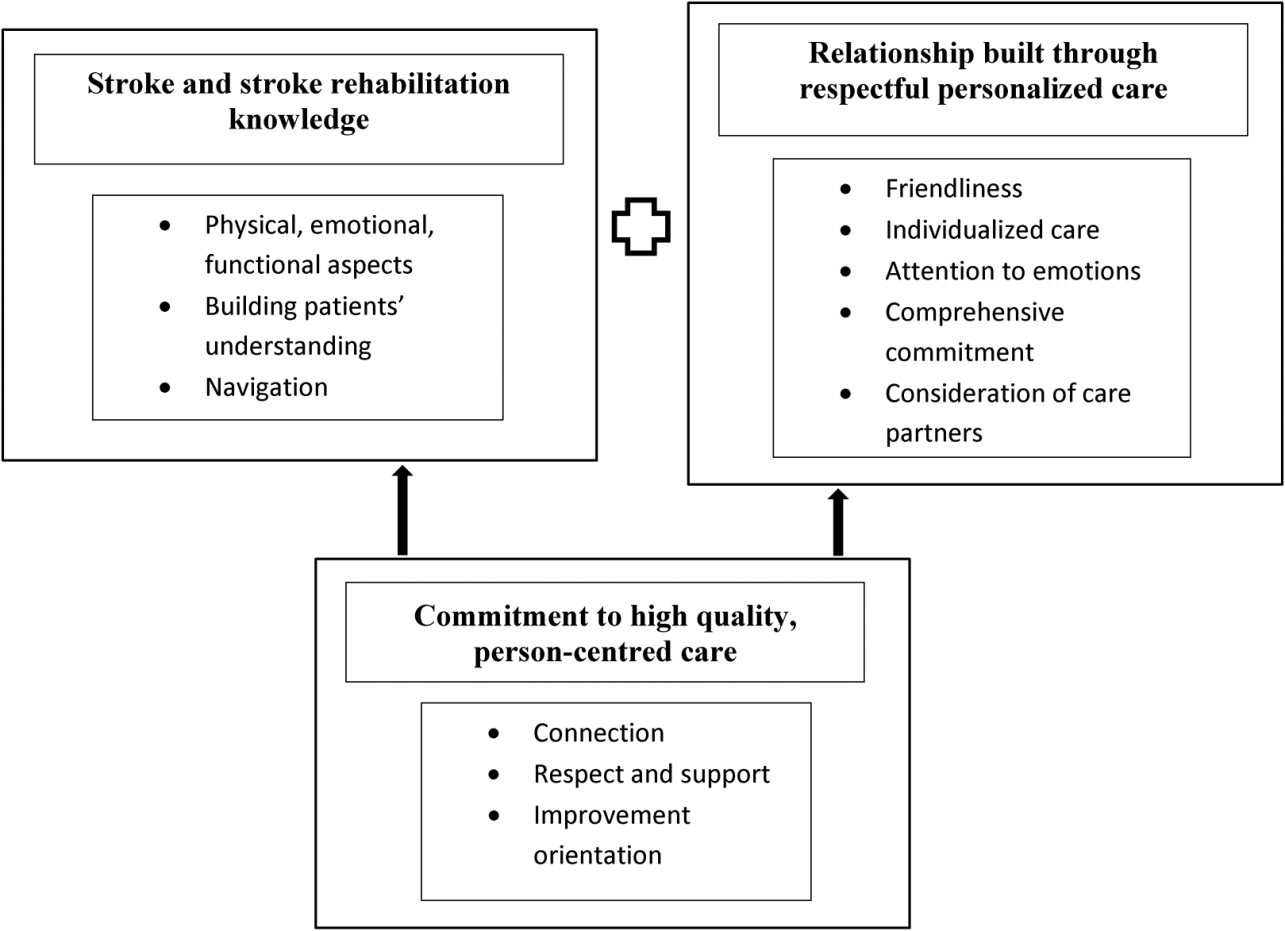
Exemplary post-discharge stroke rehabilitation.

Exemplary care was characterized by three essential components. These were: stroke and stroke rehabilitation knowledge, relationship built through respectful personalized care, and commitment to high quality person-centered care (Figure 1). These components are described below with illustrating quotes from patient, care partners, provider, and administrator participants. Patients and care partners are identified by pseudonyms.

#### Stroke and Stroke Rehabilitation Knowledge

Patients and care partners viewed providers as experts who understood and could explain the physical and emotional aspects of stroke and what could be done to improve function.

[The physiotherapist] was just very knowledgeable and very well rounded. She gave specific exercises to me in my particular level of progression… And she gave good explanations. (Peter, Program D)

Especially appreciated was explanation of stroke-related issues patients could do to address these.

I’ll do it to the best that I can, sometimes … until I have-- I come to a stop and I meltdown. I do these meltdowns where it's, like, I can't even think (Tina, patient, Program C).

That is an issue, and they’ve figured that one out too (Jenn, care partner).

Yeah, they’ve just figured that one out, yeah (Tina).

Finally, patients and care partners appreciated team members’ ability to navigate the health care system in efforts to solve complex problems. For example, Martin (patient, Program A) had a complex swallowing problem worsened by a fall. From Martin's health records, it was clear that the speech-language pathologist had spent considerable time skillfully exploring the problem and testing potential solutions, and then helping Martin access medical specialists to diagnose his complex problem so that it could be effectively addressed.

Providers shared their passion for helping patients make sense out of their experiences of stroke and accompanying body changes. They were excited to further develop their knowledge of stroke and stroke rehabilitation. Administrators expected stroke rehabilitation expertise from staff and provided multiple opportunities to maintain and improve knowledge and skills. In addition to using formal sources of education, providers regularly sought out the expertise of other members of their team.

Stroke and stroke rehabilitation expertise formed the background of exemplary care. It consisted of concrete knowledge, expertly shared and applied. The next component, relationship built through respectful personalized care, consisted of more abstract elements.

#### Relationship Built Through Respectful Personalized Care

Five factors contributed to the building of relationships: friendliness, individualized care, attention to emotions, comprehensive commitment, and consideration of care partners.

#### Friendliness

Providers got to know the patients as people, by learning about their histories, families, concerns, interests, and personalities. Patients enjoyed being with their providers and sensed this enjoyment was mutual. They felt valued and encouraged by their providers and accepted rather than judged.

I connected with these people. I think they did with me. So it was probably like having a good conversation with a friend. Good friend, you know, someone you trust. And they were very good at establishing the trust level. I felt comfortable saying what was bothering me too to them and I knew they would listen (Kevin, Program B)

There was team commitment to reflecting on and consistently acting in ways that showed this respect.

They’re welcoming us into their home, and we’re highly respectful of that. So it's just even the little things that add up. So we’ll bring in shoes, indoor shoes and change our shoes. We’re prompt with returning phone calls. Prompt with appointments. If we’re running more than 15 min late, we will call them and let them know. (Provider, Program B)

#### Individualized Care

Care was individualized according to patients’ goals, personalities, and progress. All programs had an initial process that involved getting to know the patients: understanding their goals, relationships, and valued activities. Individual patient goals were developed to guide the entire program. Formal development of patient goals explicitly included asking about valued activities within the home or community.

And I think the goal is working interdisciplinarily and helping clients get back to life, whatever that means for them. So for somebody their goal is getting up in the morning, bathing themselves and watching TV all day, well, then that's what we’ll help them get back to. For the next person it might be getting back to coffee with their friends. And so it's getting back to life, whatever that life means. (Provider, Program B)

I think just the fact that they sat there and listened and interviewed me and tailored the program, you know, everything they did met my needs. They weren't just giving me a book and saying follow these examples. It was a program for me… since you’re working on stuff that is important to me, since I was working on that sort of stuff, I think I recovered faster. (Hank, Program A)

As well, patients reported that they were regularly provided concrete feedback about their progress. Sometimes this was done through review of changes in standardized measures. Patients found this encouraging, particularly at times when they were not aware of incremental changes.

They test me regularly where I am and then they show me there's improvement. I think there was no improvement but when they show me … there was improvement, right. So you don't feel that bad, right. (Robin, Program D).

Flexibility was an essential characteristic of personalized respectful care. Each patient's care was designed, coordinated, and delivered in a flexible manner, particularly in terms of location, timing, providers, and discharge. All programs considered patient energy levels and other commitments when organizing care.

I’ll go and do my first visit and I might give them a week or two break. Just to kind of let them settle in at home. We get the referral the day that they’re discharged from the hospital. So sometimes they haven't even been home 24 h and we’re calling saying, hey, I want to come in. And they’re going, oh my god. So you can tell a little bit pretty quickly who's a little overwhelmed. And then we’ll kind of touch base quickly and go, hey, this is going to be too much for these people, what's the priority based on the referral or based on who's been in. And then we kind of pull back [in terms of intensity] in those cases. (Provider, Program A)

All programs had provisions for allowing the care to be stretched out to ensure patients received care at the best time to address specific patient goals.

Oh, what's really good about outpatient rehab is that they’re flexible with when you can come back. Let's say for my OT. I think I still have a month left [of occupational therapy] and they have helped me save that time to [work on] going back to work. So that's really good about the program. (Francis, Program D).

Across all programs, at least some elements of care were or could be provided in the home or at community locations where valued activities took place.

So the patient doesn't have to fit into this very rigidly defined box of what rehab is… it's the flexibility in terms of addressing things at home. We’ve also addressed things like if the goal is to go to a swimming program then the therapist goes to the swimming program with the patient. So I would say, yeah, it's the ability of the program to adapt to the patient's goals and needs. (Administrator, Program A)

#### Attention to Emotions

Patients noted that providers spent time checking in with them to see how they were feeling and what had been happening in their lives since the last appointment. Providers were attentive to signs of depression. They encouraged patients to talk to them about their feelings by normalizing depression. Providers pointed out that depression was common after stroke, and explained it as a “symptom,” like physical manifestations of stroke. They stressed that effective intervention was available, and gently offered them social work or other counselling services, often repeatedly. This facilitated patients’ eventual connection with these services. Environmental conditions for privacy, provided by home visits or private offices in clinic spaces, supported frank discussions between patients and providers.

[And each time I met with a provider] 15, 20 min of just talking before we went anywhere… Yeah, they knew what was bothering me and I knew …. we all had a common goal. (Hank, Program A)

Patients’ appreciative comments regarding the ease with which they could openly share their sadness, fears, and frustration were the most prominent feature of patient interviews in Programs A, B, and C and present in Program D.

They were great to talk to, you know, everybody was, you know, all the people that came were really nice and kind and, you know, so I found them, you know, it was nice to have somebody come a couple times a week and do a bit of unloading if you needed to…I mean, they were, yeah, they were all very kind and very understanding. (Sally, Program B)

While providers did not include attention to emotions in their initial descriptions of what made their programs exemplary, they did note the importance of their focus on psychosocial issues when they were told that this was a major factor for patients.

You know what I’m realizing, though, during our rounds we always start when we talk about the person about psychosocial [inaudible, voices overlap]—(Provider 1, Program A)

Yeah. We always do. (Provider 2, Program A)

Always. (Provider 3, Program A)

#### Comprehensive Commitment

Programs provided a comprehensive commitment to patients that began with program referral. Providers had difficulty recalling examples of a referred patient whom they could not serve. On very rare occasions, all services were postponed to allow patients to respond to other urgent medical or personal issues.

And I think that's such a big part of everyone that sits around the table too, is nobody gives up. So even if this client can't participate right now, the first thing that was said around the table was, when she's ready I’ll go. (Provider, Program B)

In exceptional instances where patients were referred to other programs care was taken to follow up on the referral and ensure that the patient had been accepted for these services.

This comprehensive commitment to each patient was sustained throughout the length of the program, and sometimes beyond. Care was taken to connect patients with relevant social service and community agencies to help meet basic needs, or more specialized needs.

[As the Stroke Community Navigator] I went into the home to meet with the whole family together and separately … it had gotten to the point where [patient with severe aphasia demonstrated]…aggression because they couldn't communicate [with a family member due to aphasia]. I helped connect the family member with counseling and also gave her some resources and also connected the family with an individual living with stroke. … I attended crisis counseling with the client. I also talked to a [specialized] social worker that he could go to without being able to speak … [I also] connected with Behavioural Supports Ontario, and Active Aging through the Alzheimer's Society. (Provider, Program C)

Connections with community agencies, programs, and facilities were made to support continuation of valued activities. Services were explicitly and implicitly designed to ensure that patients and care partners could manage following discharge.

…in outpatient services, here we are, the last on the chain, the last on the publicly serviced, publicly funded chain of available rehab. And here we are preparing our clients, essentially for the rest of their lives. And that can be-- that's quite a sense of responsibility that, I think, each and every one of us take very, very seriously. (Provider, Program D)

Finally, each program seemed to have a way for patients to reconnect if they needed help.

#### Consideration of Care Partners

Care partners were recognized and provided with access to support in all programs. As well, providers connected with care partners in the home or at the clinic before, during or after sessions, or booked in-person or phone family conferences.

In Program C, the stroke navigator considered both patients and care partners to be clients. She met with care partners and patients together or separately. Providing emotional support to care partners was an explicit part of her role.

Care partners expressed the need for even more services. Providers and administrators appreciated this need, and, at the time of the interviews, Program A and C were working toward offering more programming for care partners.

The five factors friendliness, individualized care, attention to emotions, comprehensive commitment, and consideration of care partners provided the foundation for a therapeutic relationship that was a key ingredient in patients’ continued commitment to working toward this recovery, their hope for continued recovery, and the construction of a good life following stroke.

And it's the relationship they build. They’re very open, accommodating people…And they create that kind of bond that it makes you feel safe and makes you want to please and progress …It gave you a sense of hope. You felt hope that somebody's out there who cares and [is] encouraging you to get stronger. And that you can progress. That it's not over. As long as you have the mindset to go further then you can. It's not impossible. That was helpful. (Laura, patient program B)

Care features related to relationship-supporting factors were prominent across the four programs and constituted the bulk of patients’ feedback in Programs A, B, and C. All program health records had documentation of each patient's goals. While stroke expertise provided the background for exemplary care, personalization provided the essence. However, description of associated actions related to personalizing care were largely missing from the health records. Notably, with the exception of social work and stroke community navigator notes, documentation of emotional support was often missing or short and nondescript.

#### Commitment to High-Quality, Person-Centered Care

Finally, the third essential aspect of exemplary was care team and administration commitment to high-quality, person-centered care. This essential aspect included connection, respect and support, and improvement orientation.

#### Connection

Program providers worked to stay connected with patients and care partners and each other. All program personnel strongly identified with the service and its commitment to patient-centered care.

It's very tailored to patients’ individual needs and it's a very close team, so we work very closely and we can talk to each other and support each other's goals. Everybody's very … patient-oriented, basically, it's not about us, it's about [them]. (Provider, Program A)

Providers were continuously connected with each other. They spoke with each other regarding changes in patient circumstances. They were alert for issues that might be better addressed by or with another provider, and they referred to each other quickly and regularly. This was done both formally during regular patient review meetings, or informally through calls and text messages. Provider communication was valued by administration.

… when you’re spread out everywhere all the time, you may not see your colleagues that much. So we had to really create - if not face-to-face then that virtual community so they’re always in touch. I think they feel very comfortable with that. (Administrator, Program A)

#### Respect and Support

Team members felt respected and supported by one another.

I think there's such a mutual respect among team members of the knowledge, the capacity and the skills and abilities of each and every one of us. So I know that if I had an issue with something or if I wasn't too sure what to do, I could go to any one of my colleagues and say to them, okay, I had a thought on this. What did you think about this? And I know that there would be collaboration with my colleagues. And I also know that oh, I’ve encountered something well beyond my scope. I need to go to somebody else to ask them to work on the situation with the client or with me with the client. (Provider, Program D).

Providers also felt administrators were supportive and attentive to their feedback regarding the program. Providers had the sense that administrators might not grant all requests, but they would always listen to them. Administrators valued the expertise and input of the providers.

I feel like it has always been that mentality of let's build this together as a team. Let's go to the team for feedback. Let's incorporate the team's ideas. Like what the team thinks we need to work on, what do we think that-- where do you think are opportunities for improvement. (Administrator, Program B)

#### Improvement Orientation

All of the exemplary teams demonstrated an improvement orientation towards their work. They seemed to be always asking questions such as how can I improve the care I provide and which additional services might the program develop to address the needs of patients and care partners.

So I think we’re just always evolving, always changing and that's also a good thing too. We’re not just-- that's enough sort of thing, right. And management allows that which is also great. (Provider, Program C)

## Discussion

Across four geographically distinct regions, each with different resources, three categories of common core features of exemplary post-discharge rehabilitation were apparent: stroke and stroke rehabilitation knowledge, relationship built through respectful personalized care, and commitment to high-quality person-centered care. The first category, stroke and stroke rehabilitation knowledge, encompassed information regarding assessments, information considered within stroke rehabilitation guidelines.^17^ However, this feature also included knowledge and skill related to how specific impairments affected daily function and what patients could do to improve daily function given these issues. As well, it encompassed skill regarding how this information could be best shared with patients. In this way, principles of adult learning^18^ and health literacy^19^ were incorporated.

Also essential to stroke and stroke rehabilitation knowledge was understanding of navigation. This included objective knowledge, such as details regarding the local community, and interpersonal and organizational skills required to obtain and coordinate these services. There have been calls to add stroke community navigators to services for patients following hospital discharge.^20^ Our results indicate that navigation can be done effectively by all team members provided that they have the requisite knowledge, skills, and support. However, in areas where low therapist concentration precludes inclusion of navigation in therapists’ roles, a dedicated stroke community navigator may be essential.

Application of an ethics of care^13^ lens allowed us to uncover essential features of exemplary post-discharge stroke rehabilitation that went beyond biomedical considerations. Such features related to personalized care and included flexibility regarding the timing of intervention, learning about the person, their life circumstances, relationships, and the activities they hoped to return to. Provision of personalized care required timing and intensity that differed from in-patient care, particularly so that care could be stretched out to allow important goals to be addressed.

In addition, personalized care was characterized by friendliness. Friendliness may seem an inappropriate term to describe a patient-provider relationship. However, patients saw providers as *similar* to good friends; they seemed to like and be interested in each patient, could be trusted by them, and were a source of valued support. Increasingly, the ability to provide respectful personalized care, and to build and maintain a therapeutic alliance, is recognized as essential to effective rehabilitation.^21,22^ Our study added support to emerging findings regarding the importance of person-centered care in neurorehabilitation. There have been increasing calls to better characterize what such care looks like in practice.^23^ Terry and Kayes, looking across patient and provider qualitative data from three studies, identified four essential features: focusing on patients’ experience and needs given the difficult new reality of living with the effects of stroke; relational care as the basis of therapy and; supportive promotion of autonomy.^24^ Terry and Kayes noted that these features were put into practice despite restrictions within the systems practitioners were working. While it may be possible to introduce such care without administrative support, sustainability may be difficult.^25^ Through the inclusion of administrators among our participants, our study adds to existing knowledge by identifying their essential role in the provision of exemplary post-discharge rehabilitation. As well as championing and supporting personalized care, administrators provided real or virtual space for meeting and tools and processes to maintain communication between team members. They were open to team concerns and expected that team members would build and improve the service. Their expectations and support reinforced the teams’ identities as skilled professionals committed to their patients and dedicated to continuous refinement of the program.

In addition to administrative support, recognition of the importance of interpersonal aspects of care in best practice guidelines may also be helpful in ensuring sustainability in practice. Currently, best practice guidelines provide little detailed information regarding the interpersonal aspects of working with patients, care partners, and other team members.^17^ This may implicitly promote the idea that the abilities to provide personalized respectful care and work collaboratively with other team members are implicit, naturally occurring features, rather than skills that requires expertise and ongoing support. Historically, and even today, such skill has been discussed as occurring naturally among members of largely female, caring professions.^26^ Feminist scholars have pointed out how this essentialist argument hides the fact that these are acquired skills that require development^27^ and maintenance.^22^

As well, lack of explicit recognition of the importance of such care can lead to health record structures that discourage its documentation,^28^ keeping important elements of care hidden. When the importance of and required skill for such care is not recognized, continuing education may focus solely on technical aspects of care. Moreover, support to sustain such care could become difficult to justify. Therefore, we highly recommend essential aspects of exemplary post-discharge care, beyond knowledge of the identification and management of impairment, be carefully outlined in clinical guideline documents.

Regarding potential differences between in-patient and post-discharge stroke rehabilitation, it appeared that individualization of care required flexibility in terms of intensity and timing. This flexibility helped ensure equitable participation of patients with medical or social issues that precluded intensive programming. It also permitted care stretched out over several months, to support longer term goals such as return to work, while keeping within the fiscal restraints of the program.

There was one main limitation to the study: patients and care partners were nominated by providers who may have selected those with the best experiences. However, as the aim of this study was to explore aspects of exemplary care, it was relevant to focus on such experiences. Notably, the study benefitted from examination of multiple sources of data. These sources allowed us to gain a deep understanding of the fundamentals of exemplary post-discharge programs across different contexts.

Our case study of four exemplary post-discharge stroke rehabilitation programs identified essential aspects of such care. Each program took provincial guidelines as a starting point and applied these to provide services relevant to regional resources and needs. While team members used knowledge related to assessment and treatment of stroke impairment, they also demonstrated expertise in relational care. Importantly, their work as a committed, connected team was supported by administration. These three aspects, stroke knowledge, relationship built through respectful personalized care, and commitment to high quality person-centered care, appear essential to exemplary post-discharge stroke rehabilitation.

**Clinical messages**

- Exemplary post-discharge stroke rehabilitation can be characterized by stroke and stroke rehabilitation knowledge, relationship built through personalized respectful care, and a commitment to high-quality, person-centered care.
- The knowledge and skills required to provide exemplary post-discharge stroke rehabilitation go beyond an understanding of evaluation and intervention considerations in current best practice guidelines.
- Administrative support is essential to the development and maintenance of exemplary post-discharge rehabilitation programs.

## Supplemental Material

sj-docx-1-cre-10.1177_02692155221144891 - Supplemental material for Exemplary post-discharge stroke rehabilitation programs: A multiple case study Click here for additional data file. Supplemental material, sj-docx-1-cre-10.1177_02692155221144891 for Exemplary post-discharge stroke rehabilitation programs: A multiple case study by Mary Egan, Debbie Laliberte Rudman, Monique Lanoix, Matthew Meyer, Elizabeth Linkewich, Phyllis Montgomery, Jenn Fearn, Beth Donnelly, Margo Collver, and Shauna Daly in Clinical Rehabilitation
