# Supplementary material for: Exemplary post-discharge stroke rehabilitation programs: A multiple case study
Source: Clin Rehabil. 2022 Dec 21;37(6):851–63. doi: 10.1177/02692155221144891 (PMC10126453; doi:10.1177/02692155221144891)
Supplement: sj-docx-1-cre-10.1177_02692155221144891 - Supplemental material for Exemplary post-discharge stroke rehabilitation programs: A multiple case study [file sj-docx-1-cre-10.1177_02692155221144891.docx]

**Supplementary material**

Supplementary Table 1. Region and program characteristics

| Program | A | B | C | D |
| --- | --- | --- | --- | --- |
| Region type | Small city with much of population in surrounding rural areas | Medium-sized city with some of population in surrounding rural areas | Small northern city with much of population in surrounding small towns | Large city |
| Proportion population rural (%) | 60 | 28 | 30 | 0 |
| Proportion population over age 65 (%) | 21.8 | 18.2 | 19.0 | 15.1 |
| Mother tongue French | 22.1 | 1.3 | 23.2 | 2.3 |
| Occupational therapists per 100,000 | 42.7 | 51.9 | 37.5 | 76.5 |
| Speech-language pathologists per 100,000 | 22.6 | 24.6 | 21.2 | 27.2 |
| Personnel | - Rapid response nurse - Occupational Therapist - Physiotherapist - Rehabilitation Assistant - Speech Language Pathologist - Social Work | - Nurse - Occupational Therapist - Physiotherapist - Rehabilitation Therapist - Social Worker - Speech Language Pathologist - Therapeutic Recreation Specialist | - Rehabilitation Support Workers - Stroke Community Navigator - Contracted Occupational Therapist and Physiotherapist | - Occupational Therapists - Occupational Therapy Assistants - Physiotherapists - Physiotherapy Assistants - Physiatrists - Psychologist - Psychometrist - Social workers - Speech Language Pathologists |
